# The accessory Sec system (SecY2A2) in *Streptococcus pneumoniae* is involved in export of pneumolysin toxin, adhesion and biofilm formation

**DOI:** 10.1016/j.micinf.2017.04.003

**Published:** 2017

**Authors:** Mikaila Bandara, J. Mark Skehel, Aras Kadioglu, Ian Collinson, Angela H. Nobbs, Ariel J. Blocker, Howard F. Jenkinson

**Affiliations:** a School of Oral and Dental Sciences, University of Bristol, Lower Maudlin Street, Bristol, BS1 2LY, UK; b School of Cellular & Molecular Medicine, University of Bristol, University Walk, Bristol, BS8 1TD, UK; c Biological Mass Spectrometry and Proteomics, MRC Laboratory of Molecular Biology, Francis Crick Avenue, Cambridge, CB2 0QH, UK; d Department of Clinical Infection, Microbiology & Immunology, Institute of Infection and Global Health, University of Liverpool, 8 West Derby Street, Liverpool L69 7BE, UK; e School of Biochemistry, University of Bristol, University Walk, Bristol, BS8 1TD, UK

**Keywords:** *SecA2*, *Streptococcus pneumoniae*, Pneumolysin, Export, *O*-glycosidase, Biofilms

## Abstract

In *Streptococcus pneumoniae* TIGR4, genes encoding a SecY2A2 accessory Sec system are present within a locus encoding a serine-rich repeat surface protein PsrP. Mutant strains deleted in *secA2* or *psrP* were deficient in biofilm formation, while the Δ*secA2* mutant was reduced in binding to airway epithelial cells. Cell wall protein (CWP) fractions from the Δ*secA2* mutant, but not from the Δ*psrP* mutant, were reduced in haemolytic (pneumolysin) activity. Contact-dependent pneumolysin (Ply) activity of wild type TIGR4 cells was ten-fold greater than that of Δ*secA2* mutant cells suggesting that Ply was not active at the Δ*secA2* cell surface. Ply protein was found to be present in the CWP fraction from the Δ*secA2* mutant, but showed aberrant electrophoretic migration indicative of protein modification. Proteomic analyses led to the discovery that the Δ*secA2* mutant CWP fraction was deficient in two glycosidases as well as other enzymes involved in carbohydrate metabolism. Taken collectively the results suggest that positioning of Ply into the cell wall compartment in active form, together with glycosyl hydrolases and adhesins, requires a functional accessory Sec system.

## 1 Introduction

*Streptococcus pneumoniae*, also known as the pneumococcus, colonizes the human respiratory tract. This can be asymptomatic, but in more susceptible individuals e.g. infants, the elderly, immunocompromised, the bacteria can move to other body sites and cause sinusitis, otitis media, pneumonia or meningitis. Pneumococci exhibit many of the properties of oral viridans streptococci, such as α-haemolysis on blood agar caused by hydrogen peroxide production, adherence to glycoproteins [1], biofilm formation [2] and natural competence for DNA-mediated transformation [3]. Components known to be essential for full virulence are the capsular polysaccharides, of which there are more than 90 serotypes [4], choline-binding proteins CbpA, LytA, PcpA, PspA and PspC [5], pore-forming toxin pneumolysin (Ply), and various cell-surface-associated proteins Eno, NanA, PavA, PavB, PsaA and PsrP [6], [7].

Ply is a highly conserved 53-kDa pore-forming toxin that is a member of a protein family known as the cholesterol-dependent cytolysins (CDCs). Members of this toxin family are expressed in *Streptococcus*, *Clostridium* and *Listeria*, and include streptolysin O, perfringolysin O and listeriolysin O. The CDC toxins bind to cholesterol in target membranes, and once inserted into the membrane they oligomerize to form pores (350–450 Å in diameter for Ply) [8], resulting in host cell lysis and tissue damage [9]. Ply is produced by virtually all clinical isolates of *S. pneumoniae* and is multifunctional. In addition to cytolytic activity, Ply modulates the immune system by activating the classical complement pathway [10] and neutrophil extracellular trap formation [11]. Ply also affects lysosomal integrity in epithelial cells [12], induces DNA damage and cell cycle arrest [13], and is reported to impact on biofilm formation [14]. However, a confounding issue about the production of Ply is that, unlike other CDC family members, Ply lacks an N-terminal leader peptide to direct secretion through the canonical Sec pathway. Consequently, it has been considered an intracellular protein, released only following cell lysis in vitro or in vivo [15]. More recently, evidence has emerged for autolysis-independent release of Ply [16], and for exported Ply to be localized mainly to the bacterial cell wall [17]. The current notion is that Ply is exported into the cell wall peptidoglycan matrix, within which branch-stem peptides act as a barrier to Ply release [18]. However, the precise mechanism for Ply export across the cell membrane has not yet been established.

Fusion of a canonical Sec signal sequence to Ply did not allow Sec-dependent Ply secretion in *S. pneumoniae*, although secretion occurred when the same construct was expressed in surrogate host *Bacillus subtilis* [19]. More importantly, *B. subtilis* secreted Ply with no leader peptide added, suggesting the existence of a conserved protein export system that is coupled to cell wall localization [19]. In *L.* *monocytogenes*, listeriolysin O is found extracellularly in complex with a putative chaperone protein (FbpA) and internalin B (InlB) [20]. FbpA protein is one of a number of leader-less proteins in *L. monocytogenes* that are secreted via an alternative pathway known as the accessory Sec system [21]. We hypothesized that the accessory Sec system in *S. pneumoniae* facilitates the export of Ply across the cytoplasmic membrane into the cell wall environment.

**Fig. 1:**
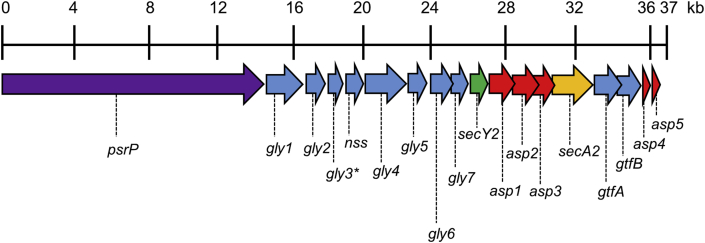
Schematic representation of the ∼36 kb accessory *secY2A2* locus derived from the genome sequence of *S. pneumoniae* TIGR4 (GenBank™ accession number AE005672.3). Genes encode the following: serine-rich repeat protein *psrP* (SP_1772); glycosyltransferases *gly1* (SP_1771), *gly2* (SP_1770), gly3* (SP_1769), *nss* (SP_1768), *gly4* (SP_1767), *gly5* (SP_1766), *gly6* (SP_1765), *gly7* (SP_1764), *gtfA* (SP_1758, SP_RS08705), *gtfB* (SP_1757, SP_RS08700); accessory secretion proteins *asp1* (SP_1762), *asp2* (SP_1761), *asp3* (SP_1760), *asp4* (SP_1756), *asp5* (SP_1755); *secY2* (SP_1763) and *secA2* (SP_1759, SP_RS08710). *gene contains a frame shift mutation.

In *S. pneumoniae* TIGR4 (serotype 4), *secY2* (transmembrane protein) and *secA2* (ATPase) genes are found within a 37-kb pathogenicity island encoding cell-surface pneumococcal serine-rich repeat protein (PsrP, 4776 aa residues) (Fig. 1). An additional 10 genes encode glycosylation enzymes, and five *asp* genes encode transport complex proteins [22], [23]. A similar locus is found in several but not all sequenced pneumococcal genomes. By analogy to genomic loci in *Streptococcus gordonii* and *Streptococcus parasanguinis* encoding serine-rich repeat (SRR) proteins GspB [24], Hsa [25], [26] and Fap1 [27], PsrP in *S. pneumoniae* is predicted to become post-translationally glycosylated concomitantly with secretion via the alternate SecY2A2/Asp1-5 translocon [28]. The C-terminus of PsrP glycoprotein is cell wall-anchored while the N-terminal region is projected away from the cell surface and binds keratin [29]. In addition, PsrP plays a role in biofilm formation in vitro [30] and in vivo [31] by mediating direct cell–cell accumulation, or indirectly through binding extracellular DNA [32].

To determine if Ply export was mediated or modulated by the SecY2A2 system in *S. pneumoniae*, the translocon function was inactivated by deletion of the *secA2* gene. The effect of this was to inhibit biofilm formation, association to lung epithelial cells, and export of Ply to the cell wall compartment. Our results suggest that the SecY2A2 translocon is required for efficient localization of Ply to the cell surface.

## 2 Materials and methods

### 2.1 Bacterial strains and growth conditions

Bacteria and plasmids used are shown in Table S1. Pneumococci were grown on THY-blood agar (3.6% Todd-Hewitt broth, 5% yeast extract, 12% agar) with 5% defibrinated horse blood, or in THY broth, at 37 °C in 5% CO_2_, supplemented as appropriate with 100 μg spectinomycin (Sp) ml^−1^, 2 μg erythromycin (Em) ml^−1^ or 2 μg chloramphenicol (Cm) ml^−1^. *Escherichia coli* was cultivated in LB medium, with 100 μg ampicillin (Ap) ml^−1^ or 300 μg Em ml^−1^ as required, and was manipulated by standard protocols. Overnight cultures (10 ml) of all bacteria were grown for approximately 16 h unless stated otherwise. For long term storage (glycerol stocks), overnight cultures were pelleted and resuspended in appropriate broth containing 15% glycerol. Strains were either placed into the culture collection or aliquoted when needed for frequent use and stored at −70 °C until used. Cultures were always inoculated with standard amounts of frozen glycerol stock suspensions.

### 2.2 Generation of mutants

To generate a *secA2* deletion, chromosomal DNA from *S. pneumoniae* TIGR4 (wild type, WT) was PCR-amplified (Expand Long Template, Roche Diagnostics, Mannheim, Germany) with primer pairs MBF7/MBR7 and MBF8/MBR8 (Table S2), producing 552-bp and 579-bp *secA2*-flanking regions. These were ligated by PCR, generating a central BamHI site, and cloned into pGEM-T in *E. coli* JM109. Spectinomycin-resistance cassette *aad9* (1015 bp) was PCR-amplified from pFW5 [33] with primer pair BamSpecF/BamSpecR (Table S2) and cloned into the unique BamHI site within the flanking regions construct. This was purified, the insert was excised with SacII and SpeI, gel-purified and transformed [34] into *S. pneumoniae* TIGR4 selecting for Sp^R^. Fidelity of strain UB2570 Δ*secA2* was confirmed by PCR-amplification with MBF7/MBR8 primers and DNA sequencing. A similar method was employed to produce a Δ*ply* mutant. Flanking regions of *ply* (624 bp and 940 bp) were PCR-amplified with primer pairs Up.PlyF/Up.PlyR and Down.PlyF/Down.PlyR (Table S2), ligated by PCR and cloned into pGEM-T in *E. coli* DH5α. Chloramphenicol resistance cassette *cat* (937 bp) was amplified from pR326 [35] with primers BamCatF2/BamCatR, cloned into the unique BamHI site within the flanking regions plasmid construct, and then excised (2513 bp), purified and transformed into *S. pneumoniae* as described above, selecting for Cm^R^. Strain UB2706 Δ*ply* was confirmed by PCR with Up.PlyF/Down.PlyR and DNA sequencing. To generate a double mutant, the 2513-bp Δ*ply*::*cat* fragment was transformed into UB2570 with selection for Cm^R^ and Sp^R^. Strain UB2707 Δ*secA2*Δ*ply* was confirmed by PCR and DNA sequencing as before.

Complementation of *ply* in strains UB2706 and UB2707 was achieved as follows. The *ply* gene (1416 bp) was PCR-amplified using primer pair Nco1PlyF/Xho1PlyR (Table S2). Expression plasmid pMSP7517 [36], with nisin-inducible promoter, together with *ply* fragment, were digested with NcoI and XhoI restriction enzymes, ligated and transformed into *E. coli* DH5α selecting Em^R^ (300 μg ml^−1^). The resulting pMSP*ply* (denoted p*ply*) was then transformed into UB2706 or UB2707 selecting Em^R^, generating UB2717 Δ*ply*/p*ply* and UB2719 Δ*secA2*Δ*ply*/p*ply*. Expression of *ply* was induced with 10 ng nisin ml^−1^.

For mutagenesis of Thr_63_ to Ala, p*ply* (10.116 kb) was PCR-amplified (Phusion^®^, New England Biolabs, Ipswich, MA) with primer pair PlyMutA.F/PlyMutA.R (Table S2) such that GCC (Ala codon) on primer PlyMutA.F replaced ACA (Thr) present in *ply*. The re-ligated plasmid was transformed into *E. coli* DH5α, purified, then transformed into pneumococcal strains UB2706 and UB2707 to generate UB2768 Δ*ply*/p*ply*_63_ and UB2769 Δ*secA2*Δ*ply*/p*ply*_63_. All strains were confirmed by appropriate PCR amplifications and DNA sequencing of PCR fragments.

### 2.3 Phenotypic assays

#### 2.3.1 Growth curve

*S. pneumoniae* strains were grown for 9 h in THY medium at 37 °C. Overnight cultures (250 μl) were sub-cultured into pre-warmed fresh broth (10 ml). *A*_600_ of the bacterial suspensions were measured at 1 h intervals until cultures reached stationary phase (≤9 h).

#### 2.3.2 Biofilms

Biofilm assays were performed as previously described [26]. Sterile coverslips (19 mm diameter) were placed in each well of a 12-well polystyrene tissue culture plate (Greiner Bio-One), and 0.5 ml of 10% saliva [37] was added. Plates were incubated at 4 °C overnight. Overnight cultures were equilibrated to *A*_600_ = 0.1 with fresh broth. Saliva was removed from coverslips, and portions (0.5 ml) of culture was added to wells containing saliva-coated coverslips in quadruplicate for each strain. Biofilms were grown for 6 h anaerobically at 37 °C. Media were removed, and coverslips were rinsed in PBS. Biofilms were stained with 0.5% crystal violet (1 ml) for 15 min, washed with distilled H_2_O until excess stain was removed, and air-dried. For visualization of biofilms, coverslips were inverted and mounted onto microscope slides and viewed on a light microscope (Leica, Milton Keynes, UK) with attached colour view camera and images captured using CellD imaging software (Olympus, Southend-on-Sea, UK). For biomass quantification of biofilms, crystal violet was dissolved in 10% (v/v) acetic acid for 15 min, and 100 μl portions transferred to a microtiter plate (MTP). Absorbance at 595 nm (*A*_595_) was then measured on an iMark™ MTP reader (Bio-Rad). All studies were performed in triplicate, and mean biomass calculated from three independent experiments.

#### 2.3.3 A549 association assay

A549 cells (lung pneumocytes) were maintained in DMEM (Dulbecco's Modified Eagle's Medium-F-12) supplemented with 10% foetal calf serum, 2 mM l-glutamine, 100 I.U. penicillin ml^−1^ and 100 μg streptomycin ml^−1^. Cells were grown to ≥80% confluence in 75 cm^2^ flasks and collected twice-weekly by trypsin–EDTA treatment. Detached cells were seeded into fresh flasks (10^6^ cells per flask) and incubated for ∼3 d at 37 °C in humidified air-5% CO_2_. Association of *S. pneumoniae*, which encompasses both adhesion and invasion, to epithelial cells was determined by viable count assay [38]. Briefly, confluent monolayers of A549 cells in 24-well tissue culture plates were incubated with 1 × 10^6^ bacterial cells, in triplicate, in serum-free DMEM for 2 h at 37 °C in humidified air. Monolayers were washed three times to remove non-adhering bacteria, and epithelial cells were detached by adding ice-cold sterile dH_2_O (20 min). Numbers of bacteria associated with A549 cells were determined as colony forming units (CFU) from agar plate dilution counts. Monolayers were visualised on an inverted microscope (Olympus, Southend-on-Sea, UK). Light micrographs were taken at each stage of the assay to monitor the state of the epithelial monolayer to ensure that 2 h of bacterial incubation and washing procedures did not affect pneumocyte cell layer integrity (Fig. S1).

#### 2.3.4 Haemolytic assays

Haemolytic activity was measured as described elsewhere [17] with some modifications. Pneumococci in mid-exponential growth phase were harvested by centrifugation (10,000 × *g*, 10 min) washed with phosphate-buffered saline (PBS; pH 7.2), and suspended (∼2 × 10^10^ cells ml^−1^) in 350 μl cell wall digestion buffer (30% sucrose in 10 mM Tris–HCl pH 7.0, containing Sigma protease inhibitor cocktail, 1 mg lysozyme ml^−1^ and 1333 U mutanolysin ml^−1^). Suspensions were shaken gently for 3 h at 37 °C. Spheroplasts were sedimented by centrifugation (17,000 × *g*, 10 min) and supernatants containing cell wall proteins (CWPs) were retained. Spheroplasts were then lysed in 50 mM Tris–HCl pH 7.5, centrifuged as before, and the supernatant designated cytoplasmic protein (CP) fraction. Protein fractions were serially diluted two-fold in PBS containing 0.1% BSA and 10 mM dithiothreitol in 96-well U bottom plates. Suspensions of washed 2% sheep red blood cells were added (50 μl per well) and after 1 h at 37 °C the plates were visually assessed for haemolysis, then centrifuged (233 × *g* for 10 min), and absorbance of supernatants at 490 nm (*A*_490_) were measured.

To assay for contact-dependent haemolysis, *S. pneumoniae* cell suspensions in PBS (∼2 × 10^9^ cells ml^−1^) were mixed with equal volumes of 25% sheep red blood cells, centrifuged at 9600 × *g* for 5 min at 4 °C to form close contacts, and incubated for 3 h at 37 °C. Non-contact controls (no centrifugation) were included. To control for centrifugation-induced lysis of *S. pneumoniae*, bacterial suspensions alone were centrifuged as above, then mixed with erythrocytes and incubated for 3 h at 37 °C. PBS controls were also included, and all assays were run in triplicate. To determine haemolytic activities, suspensions were centrifuged (9600 × *g*, 1 min, 4 °C) and *A*_490_ of supernatants measured.

### 2.4 Protein analysis

#### 2.4.1 Cell fractionation

Bacteria were collected from 16 h cultures by centrifugation (5000 × *g*, 7 min) and identical cell concentrations were used in all extractions when comparing wild type and mutants. Pellets were suspended in TE buffer and disrupted with glass beads in a Precellys homogenizer (2 cycles of 5000 × *g* for 30 s, repeated twice with cooling on ice). Cryovials containing homogenates were pulse-centrifuged to sediment beads and debris, and the cloudy supernatants were retained at −20 °C as whole cell lysates. For fractionation of CWPs and cytoplasmic proteins (CPs) for SDS-PAGE, bacterial cell pellets were suspended in 20 mM Tris–HCl pH 6.8, containing 26% raffinose, 10 mM MgCl_2_, 0.4 mM phenylmethylsulfonyl fluoride and mutanolysin (500 U ml^−1^) and incubated for 15 min at 37 °C, centrifuged (17,000 × *g*, 15 min), and the supernatant (CWP fraction) was retained. The pellets were then vortex-mixed with 0.1 mm diameter glass beads to disrupt the cells, centrifuged (17,000 × *g*, 5 min) and the supernatant (CP fraction) was collected.

To obtain culture fluid (CF) proteins, overnight cultures of *S. pneumoniae* were sub-cultured in minimal medium TYG (0.5% tryptone, 0.5% yeast extract, 0.5% glucose, 4 g l^−1^ K_2_HPO_4_, pH.7.5) to mid-exponential phase to the same *A*_600_, pelleted by centrifugation, and the supernatant was filtered (0.22 μm pore), mixed with trichloroacetic acid (10% final concentration) and incubated on ice for 16 h. Precipitated proteins were recovered by centrifugation (20,000 × *g*, 30 min, 4 °C), washed three times with acetone, and stored at −20 °C.

#### 2.4.2 Electrophoresis and immunoblot analyses

Protein samples were heated at 80 °C for 10 min in 20 mM Tris–HCl pH 6.8 containing 1% sodium dodecyl sulphate (SDS) and 10 mM dithiothreitol, mixed with loading dye and separated by SDS-PAGE at 120 V for 1 h. M_r_ markers were PageRuler Plus Prestained protein ladder (New England Biolabs). Proteins were transferred onto nitrocellulose membrane (113 V, 1 h) and membranes were blocked with 5% milk in TBS (1 mM Tris–HCl pH 8.0, 0.15 M NaCl) containing 0.05% Tween 20 (TBS-T) overnight at 4 °C. Membranes were washed with TBS-T, incubated with 1:1000-diluted anti-Ply monoclonal antibody (Pierce Antibodies, Thermo Fisher Scientific, Waltham, MA), and bound antibody was detected with 1:2000-diluted anti-mouse IgG HRP conjugated secondary antibody (Dako, Agilent Technologies, Santa Clara, CA) followed by enhanced chemiluminescence (Biological Industries, Beit Haemek, Israel).

#### 2.4.3 Immunoprecipitation

CWP fraction (600 μg protein) was mixed with 15 μg anti-Ply antibody and incubated for 2 h with mixing. Negative controls contained no antibody. Magnetic beads (Pierce Protein A/G, 400 μg) were added together with TBS-T buffer, mixed gently, and the magnetic beads were collected to the tube sides. The suspension was removed, beads washed with TBS-T and magnetically-collected as before, and repeated twice. The antigen–antibody mixture was added to the pre-washed magnetic beads and incubated for 1 h with mixing. Ply was eluted by mixing the beads with 0.1 M glycine pH 2.0 for 10 min. The beads were magnetically separated and the supernatant was neutralized using 1 M Tris. Protein was subjected to SDS-PAGE and immunoblot analysis with anti-Ply antibody (see above).

#### 2.4.4 Mass spectrometry

Briefly, for identifying total proteins, samples were subjected to SDS-PAGE for 10 min, and the top 2 cm of the gel was excised and fixed in 7% acetic acid-50% methanol for 30 min. Gel slices as above, or of Coomassie blue-stained single bands, underwent automated in-gel tryptic digestion (ProGest, Genomic Solutions, Ann Arbor, MI). For total proteins, peptides were analysed by reverse phase nano-liquid chromatography MS/MS using a LTQ-Orbitrap Velos (Thermo Fisher) mass spectrometer. The mass spectral data for each experiment were combined prior to database searching using an in-house Mascot server to identify proteins present. Data from the Orbitrap were received in Excel and further analysis was performed locally. The identified peptide data included accession number, sequence length of the protein, molecular mass, calculated pI, a description, coverage (percentage of the protein sequence covered by identified peptides), score (total score of the protein being the sum of the scores of the individual peptides), peptide spectrum matches (total number of identified peptide sequences for the protein, including those redundantly identified), and peptides (number of unique peptide sequences). When comparing between each sample, proteins detected in the sample with a spectral count of at least three was considered a true hit.

### 2.5 Statistics

All data are reported as mean ± standard deviation (SD) of at least two independent experiments. Significance between samples was determined using the paired two-tailed Student's *t*-test, and *P* values of <0.1 and <0.05 were considered statistically significant.

## 3 Results

### 3.1 Phenotype of *secA2* mutant

**Fig. 2:**
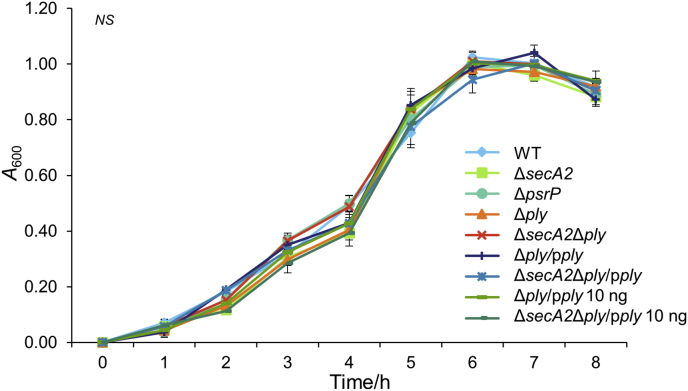
Growth curves for *S. pneumoniae* WT, mutants and complemented strains. Growth rate analyses of *S. pneumoniae* wild type, Δ*secA2*, Δ*psrP* and Δ*ply* mutant strains were carried out to assess if inactivation of these genes affected bacterial growth that might influence subsequent assays. Complemented *ply* strains were induced with 0 and 10 ng ml^−1^ of nisin as mentioned in graph legend to show that addition of nisin had no effect on bacterial growth. Strains were grown in THY medium until cultures reached stationary phase. There are no significant differences (NS) between the WT, mutants, and nisin induced complemented strains in growth rates or growth yield. (*n* = 2).

**Fig. 3:**
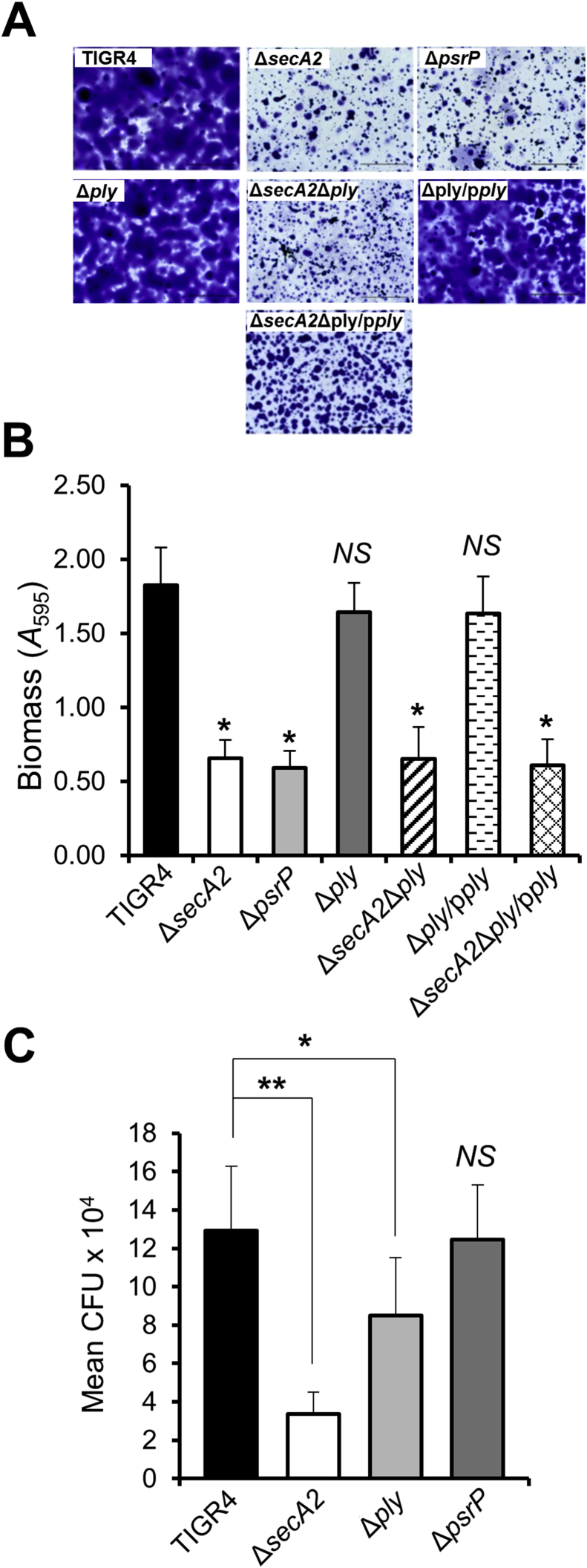
Biofilm formation and association to lung cells by *S. pneumoniae* TIGR4, mutants and *ply* complemented strains. (A) Representative light micrographs of corresponding *S. pneumoniae* biofilms grown at 6 h and stained with crystal violet. Scale bar = 50 μm. (B) *S. pneumoniae* monospecies biofilms were grown on saliva coated cover slips for 6 h at 37 °C. Total biomass was quantified by crystal violet staining as described in Materials and Methods. Statistical significance compared to the wild type TIGR4 is indicated by an asterisk (*P < 0.05). No statistical significant difference from the wild type (*NS*) is also indicated. (*n* = 3). (C) *S. pneumoniae* association with A549 epithelial cells. A549 cells were grown to a confluence of 2 × 10^5^ cells and infected with *S. pneumoniae* TIGR4 and mutant strains (1 × 10^6^ cells ml^−1^ input) for 2 h at 37 °C. After removal of non-associated bacteria and washing of the epithelial cells, the numbers of bacterial cells associated with A549 cells were determined (CFU) by agar plate counts. *P* values: * <0.1, ** <0.05. No statistical significance to the wild type (*NS*) is also indicated. (*n* = 2).

*S. pneumoniae* TIGR4 Δ*secA2* mutant was generated by allelic exchange of *secA2* with *aad9*. Strain UB2570 Δ*secA2* cells showed no detectable differences in chain length, auto-aggregation or growth rate compared to wild type TIGR4 (Fig. 2). Biofilms formed by strains TIGR4, UB2570 Δ*secA2* and UB2312 Δ*psrP*, the latter abrogated in SRR glycoprotein PsrP, were compared morphologically and quantitatively. There was approximately 60% reduced biomass for the Δ*secA2* and Δ*psrP* mutant strains compared to WT (Fig. 3A and B). We also generated a Δ*ply* mutant for haemolytic assay controls (see below) and, by contrast, the Δ*ply* mutant and complemented mutant strain produced biofilms of similar morphology and biomass to WT (Fig. 3A and B). Biofilm formation was reduced by 60% in the Δ*secA2*Δ*ply* mutant and Δ*secA2*Δ*ply*/p*ply* complemented strains (Fig. 3A and B). Therefore, under the conditions tested, expression of *secA2* (or *psrP*), but not of *ply*, was necessary for normal biofilm formation.

Since PsrP binds to keratin 10 present on lung cells [29], [39] it was hypothesized that *S. pneumoniae* Δ*secA2* and Δ*psrP* mutants would show decreased association to A549 type II pneumocyte monolayers. There was a statistically significant 70% decrease in association for Δ*secA2* mutant cells. However, the Δ*psrP* mutant exhibited similar interaction levels to WT (Fig. 3C). Thus, PsrP is not required for association to pneumocytes. Deletion of *ply* resulted in a small reduction (35%) in association (Fig. 3C) and therefore *ply* may be required for optimal association of *S. pneumoniae* to pneumocytes.

### 3.2 *secA2* expression and Ply export

**Fig. 4:**
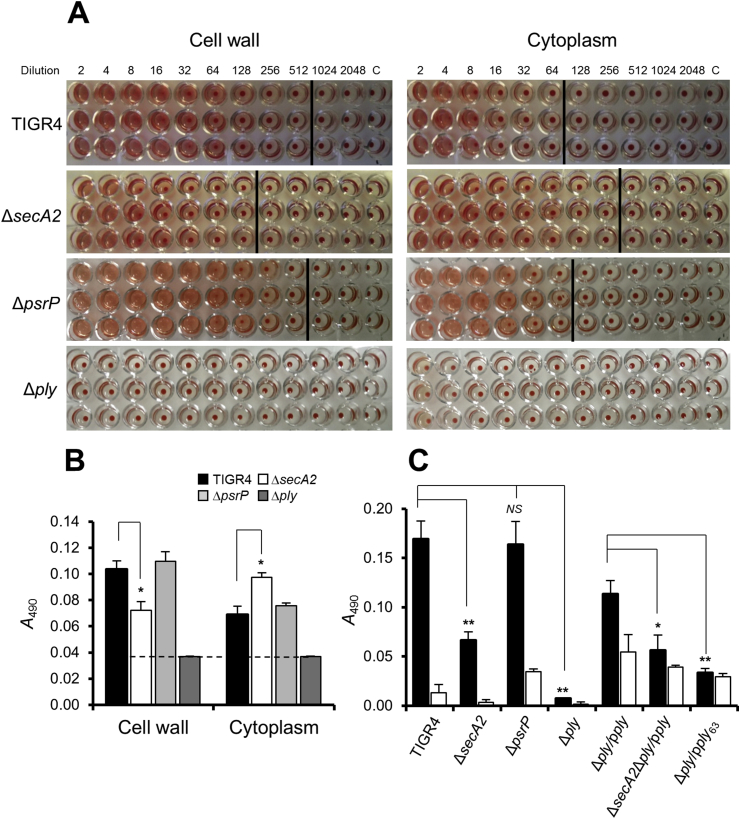
Haemolytic activities of *S. pneumoniae* wild type and mutants strains. (A) Bacterial cells were fractionated into cell wall or cytoplasm fractions (as indicated) and two-fold serial dilutions were incubated with 2% sheep RBCs in replicates of three. The control was assay buffer only. No pellet was considered to be 100% lysis. Vertical lines show the detection endpoints of 50% lysis. (B) Quantitative haemolytic data of the absorbance (*A*_490_) values of the first 50% lysis endpoint and the corresponding WT or mutant absorbance value at the same dilution. Therefore, absorbance values for cell wall and cytoplasmic fractions were taken at dilution factors 128 and 64, respectively. The dotted line represents the limit of detection. *P < 0.05. (*n* = 3). (C) Haemolytic activities of *S. pneumoniae* TIGR4, mutants and complemented strains after contact (sedimentation with sheep RBCs; black bars) or non-contact (incubation with sheep RBCs without sedimentation; white bars). *P* values: * <0.1, ** <0.05. No statistical significance to the wild type (*NS*) is also indicated. (*n* = 3).

To determine if SecY2A2 was involved in Ply export we measured haemolytic activities present in CWP or CP fractions prepared from WT or Δ*secA2* mutant strains. Haemolytic activities were present in both fractions (Fig. 4A) but the haemolysis titre for the Δ*secA2* mutant CWP fraction was reproducibly two- to four-fold lower (Fig. 4A). Correspondingly, haemolytic activity within the CP fraction of the Δ*secA2* mutant was greater than WT (Fig. 4A), suggesting a decrease in export. The Δ*ply* mutant showed no haemolysis while the Δ*psrP* mutant was similar to WT. Quantitative haemolytic assays demonstrated that Ply activity in the Δ*secA2* mutant CWP fraction was 50% lower than WT, taking into account the Δ*ply* negative control background and Ply activity in the Δ*secA2* mutant was 50% more in the CP fraction than the WT (Fig. 4B). Haemolytic activities for the Δ*psrP* mutant fractions were similar to wild type (Fig. 4B). Therefore, cell wall-associated Ply (Ply_w_) activity appears to be associated with SecY2A2 function, but is not dependent upon secretion of PsrP.

Ply_w_ activity was also determined by contact-dependent haemolysis (CDH) assay. Brief centrifugation was employed to achieve close contact between erythrocytes (sheep RBCs) and bacterial cells, compared with simply gentle mixing (designated non-contact). Appropriate controls to correct for lysis of RBCs or bacteria were included (see Materials and Methods). CDH (Ply_w_) was ten-fold higher than the non-contact activity in TIGR4 wild type (Fig. 4C). There was 60% decrease in CDH by Δ*secA2* mutant Ply_w_ compared to wild type (Fig. 4C), while the Δ*psrP* mutant was unaffected, and the Δ*ply* mutant was negative. Complemented *ply* mutant strains Δ*ply*/p*ply* and Δ*secA2*Δ*ply*/p*ply* were also included to validate the involvement of *secA2* in Ply_w_. There was >90% reduction in Ply_w_ activity in the Δ*secA2*Δ*ply*/p*ply* strain compared to the Δ*ply*/p*ply* strain, following subtraction of the activity attributable to non-contact haemolysis (Fig. 4C). The complemented strain Δ*ply*/p*ply* had lower Ply_w_ activity than wild type, and higher non-contact activity, because the cells are weakened by nisin added to induce *ply* expression.

### 3.3 Post-translational modification of Ply

**Fig. 5:**
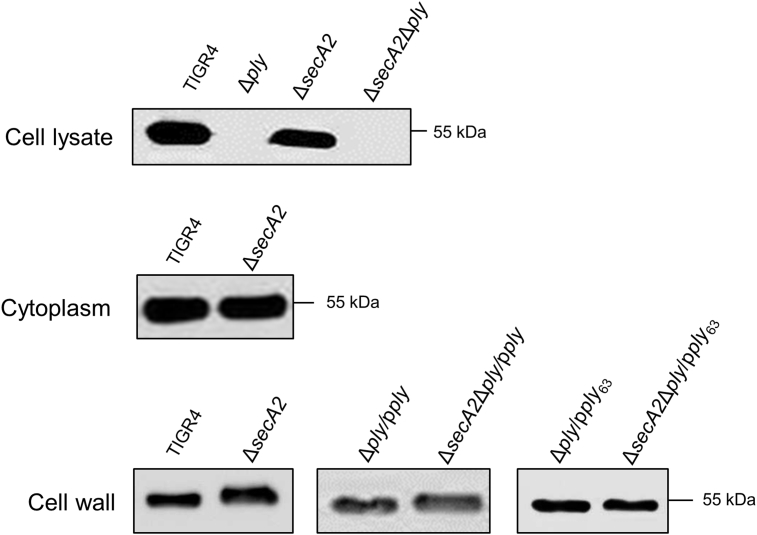
Western blot analysis of whole cell lysates and fractionated *S. pneumoniae* TIGR4, mutants and complement strains. Proteins were blotted onto nitrocellulose, reacted with anti-Ply antibody (1:1000 dilution) followed by anti-mouse HRP secondary antibody (1:2000). TIGR4 Ply is 53 kDa.

Since SecY2A2 is reported to secrete partially-glycosylated SRR proteins [24], [27], [28] we hypothesized that Ply might be post-translationally modified for export, thus explaining reduced Ply_w_ activity in the Δ*secA2* mutant. Accordingly, whole cell lysates, CWP and CP fractions from WT and mutants were subjected to SDS-PAGE and immunoblot analysis with anti-Ply antibody. In Δ*ply* mutant whole cell lysates, 53-kDa Ply expression is ablated, thus validating the Ply-specific antibody (Fig. 5). Antibody-reactive bands were present in CP and in CWP fractions from WT and Δ*secA2* mutant (Fig. 5). No significant differences in relative quantities of Ply could be seen in these fractions between WT and Δ*secA2* mutant. However, Ply_w_ in the Δ*secA2* mutant CWP fraction migrated slower by ∼2 kDa compared to WT (Fig. 5). Ply_w_ also migrated more slowly in the CWP fraction of Δ*secA2*Δ*ply*/p*ply* complemented strain compared to the Δ*ply*/p*ply* strain.

It was hypothesized that this difference might be associated with glycosylation of Ply. To investigate this, Ply was immunoprecipitated and purified from CWP fractions of TIGR4 and Δ*secA2* strains (see Materials and Methods) and subjected to SDS-PAGE and lectin-blot analysis. However, we could detect no reactivity with lectins PNA, RCA, UEA1, DBA, WGA, or SBA, variously specific to galactose, *N*-acetylgalactosamine (GalNAc), fucose, *N*-acetylglucosamine (GlcNAc) or mannose (data not shown). We then searched for potential *O*-, *N*- or *C*-linked glycosylation sites in Ply [40] and the most probable predicted *O*-linked glycosylation site was threonine residue 63 (Thr_63_) on the protein surface (Fig. S2). To determine if Thr_63_ was important for Ply_w_ localization or activity, Thr was changed to Ala by site-directed mutagenesis of plasmid pMSP*ply*. In CDH assays, there was 70% reduction in Ply_w_ activity of *S. pneumoniae* Δ*ply*/p*ply*_63_ compared with Δ*ply*/p*ply* strain suggesting that Thr_63_ was important for Ply activity (Fig. 4C). However, the apparent M_r_ of Ply_63_ in CWP fractions from UB2769 Δ*secA2*Δ*ply*/p*ply*_63_ was similar to that in the Δ*secA2* mutant (Fig. 5). Ply_w_ protein bands from WT and Δ*secA2* mutant were then subjected to in-gel digestion and LC-MS/MS as described previously [26]. There were no differences in distribution of peptides or sequences obtained from the protein samples (data not shown). Therefore, the difference in SDS-PAGE migration of Ply_w_ in Δ*secA2* mutants remains unexplained.

### 3.4 Specificity of SecY2A2

In light of evidence that multiple secreted proteins are SecA2 pathway-dependent in *L. monocytogenes* [21], we undertook proteomic analysis of CWP fractions (see Fig. S3) and extracellular proteins from WT and Δ*secA2* mutant (see Tables S3 and S4). It was clear from these comparisons that the *secA2* deletion had pleiotropic effects. While most proteins were present in similar quantities (numbers of unique peptide sequences) in WT and Δ*secA2* CWP fractions, there were several major differences. Two cell wall-anchored glycosidases (Q2MGH6 and Q97S90) were >90% reduced in the Δ*secA2* mutant, as well as a range of other proteins involved in sugar or cell wall metabolism e.g. galactokinase, M20 peptidase, glucuronyl hydrolase, as listed in Table 1. None of these were encoded by genes within the *secY2A2* locus (Fig. 1). Other proteins of interest that could not be detected in the Δ*secA2* CWP extract were RrgC (ancillary pilus subunit) and Q97NT9, which has been implicated in transport of Ply from cytoplasm to cell wall compartment [41]. In the culture fluid (CF), a number of the proteins identified in both WT and mutant e.g. EF-Tu, GAPDH, PK are also found in extracellular vesicles [42]. However, the levels of ZmpC, PcpA, Eng and AgaS were >50% reduced in the Δ*secA2* mutant CF (Table S4).

**Table 1:** Top 20 protein hits identified in *S. pneumoniae* TIGR4 CWP extracts that were <90% represented in corresponding Δ*secA2* mutant CWP extracts, compiled from data presented in Table S3. TIGR4 peptides are sorted highest to lowest number of unique peptide sequences.

Acc.^a^ (Gene name)	pI^b^	Description	% Coverage^c^		PSM^d^		Peptides^e^	
			WT	Δ*secA2*	WT	Δ*secA2*	WT	Δ*secA2*
Q2MGH6 (SP_0368)	6.07	Endo-α-*N*-acetylgalactosaminidase (Eng)	63.95	2.21	273	2	90	2
Q97S90 (SP_0498)	5.31	Putative endo-β-*N*-acetylglucosaminidase	33.33	3.92	76	3	39	3
Q97NZ6 (SP_1853)	5.08	Galactokinase	53.57	5.61	32	1	14	1
Q97NA0 (SP_2153)	4.75	Dipeptidase (M20/25/40 family)	47.63	3.84	25	1	14	1
Q97NA2 (SP_2151)	5.03	Carbamate kinase (ArcC)	61.90	4.76	41	1	13	1
Q97SL0 (SP_0322)	5.43	Glucuronyl hydrolase	37.63	0	18	0	11	0
Q97SL2 (SP_0320)	5.47	Oxidoreductase, short chain dehydrogenase/reductase family	54.61	0	25	0	10	0
P0CB58 (SP_0334)	6.76	Ribosomal RNA small subunit methyltransferase H	25.00	0	6	0	5	0
Q97P42 (SP_1802)	9.69	Uncharacterized membrane protein (sugar-binding transcriptional regulator)	23.68	0	6	0	5	0
Q97SC1 (SP_0464)	6.05	Cell surface anchor family protein RrgC (ancillary pilus subunit)	13.23	0	9	0	5	0
P0A4M7 (SP_1890)	9.10	Oligopeptide transport system permease protein AmiC	8.84	0	6	0	4	0
Q97NT9 (SP_1924)	8.98	Putative uncharacterized protein (*ply* locus)	33.09	0	5	0	3	0
Q97QK2 (SP_1203)	5.07	Arginine repressor (ArgR2)	29.37	0	3	0	3	0
Q97PP3 (SP_1560)	8.02	Putative uncharacterized lipoprotein (YbbR-like)	23.17	0	3	0	3	0
Q97NS3 (SP_0775)	4.82	Ribosomal protein S16	19.73	0	5	0	3	0
Q97S82 (SP_0506)	9.63	Integrase/recombinase, phage integrase family	13.21	0	3	0	3	0
Q97Q64 (SP_1366)	6.11	Glycosyl transferase, group 1	12.53	0	5	0	3	0
Q97T27 (SP_0129)	4.93	Probable tRNA threonylcarbamoyladenosine biosynthesis protein (Gcp)	11.61	0	3	0	3	0
Q97NZ7 (SP_1852)	5.36	Galactose-1-phosphate uridylyltransferase 2	9.74	0	10	0	3	0
Q97SL4 (SP_0318)	5.81	Carbohydrate kinase (PfkB family)	8.71	0	3	0	3	0
Q97P71^f^ (SP_1772)	4.06	Cell wall surface anchor family protein PsrP	3.10	0	32	0	14	0

a Protein accession number (gene locus).

b Calculated isoelectric point.

c Percent of the protein sequence covered by identified peptides.

d Total number of identified peptide sequences (peptide spectrum matches) for the protein.

e Number of unique peptide sequences.

f Found in unfiltered data.

## 4 Discussion

The streptococcal accessory Sec systems function to secrete SRR glycoproteins. However, the mechanism by which SRR proteins are directed to the accessory Sec system is not fully understood. The SRR protein signal (leader) peptides do not contain a YSIRK-G/S motif, which is characteristic of cell wall-anchored proteins translocated via canonical Sec [43], and the leader peptides are unusually large e.g. 88–90 aa residues. These features may reduce the affinity of SRR proteins for canonical Sec. In the streptococcal accessory *sec* loci, *gtfA* and *gtfB* genes are located immediately downstream of the gene encoding the SecA2 protein (Fig. 1). The genes encode a GtfAB (glycosyltransferase) complex, which engages with and glycosylates the pre-protein [28]. In *S. gordonii*, GspB is glycosylated first with GlcNAc, then with other sugar residues, prior to or concomitant with SecY2A2-mediated transport across the cytoplasmic membrane [44]. Potentially therefore, proteins that are translocated via the accessory Sec need to be *O*-glycosylated. In the Δ*secA2* mutant generated here, *aad9* insertion may have affected expression of downstream *gtfA*, *gtfB*, *asp4* and *asp5* genes since *aad9* carried a potential transcriptional terminator stem loop.

The accessory Sec system appears also to be involved in the export and/or activation of Ply. The Δ*secA2* mutant cell surface is depleted of haemolytic activity (CDH) while the CWP fraction, obtained by enzymatic hydrolysis of the cell wall, is ∼50% reduced in haemolytic activity. The Δ*secA2* mutant CWP fraction contains Ply_w_ protein but this displayed marginally slower electrophoretic mobility than WT Ply_w_. This suggests that in the Δ*secA2* mutant cell wall compartment, Ply_w_ is present in a modified and less active form. Our data suggest the accessory Sec system is indirectly involved in Ply export because if it were directly involved we'd expect to see a 100% decrease in Ply export to the cell wall of the Δ*secA2* mutant.

Although we have been unable at this stage to demonstrate that Ply is glycosylated, the absence of two glycosidases in the Δ*secA2* mutant cell wall would be expected to impact on glycoprotein processing and on cell wall metabolism. Clearly Ply_w_ is intimately associated with cell wall structure, since enzymatic digestion of peptidoglycan is necessary to liberate Ply from the cell wall [18].

The Δ*secA2* mutant cell walls were >90% deficient in two glycosidases, endo-α-*N*-acetylgalactosaminidase and endo-β-*N*-acetylglucosaminidase. The former, designated Eng [45], catalyses the release of Galβ1-3GalNAcα1 linked to serine or threonine residues found on *O*-linked glycoproteins. These enzymes are involved in hydrolysing oligo- or polysaccharides present in the environment, releasing sugars for bacterial cell growth, or exposing new oligosaccharide receptors for adhesion to host cells [45]. Eng has been shown to be necessary for pneumococcal binding to A549 cells, which may be a reason for why there was lower A549 association levels for the Δ*secA2* mutant. However, contrary to other work [39] we found that the Δ*psrP* mutant was unaffected in adherence to A549 cells. The glycosidases both carry predicted leader peptides (37–38 aa residues) with YSIRK-G/S motifs, and cell wall-anchorage LPXTG motifs, suggesting export via canonical Sec [43]. Although there is evidence for functional cross-talk between canonical Sec and SecA2 [26], [46], it was nonetheless surprising to find amounts of these proteins >90% reduced in Δ*secA2* mutant cell walls. The mechanism for this is currently under investigation.

Other main components present in the WT CWP fraction and >90% reduced in the Δ*secA2* mutant were metabolic enzymes without predicted leader peptides. These were: galactokinase, converting galactose to galactose 1-phosphate in the Leloir pathway; dipeptidase M20/25/40, a potential virulence factor [47]; carbamate kinase involved in anaerobic arginine metabolism; glucuronyl hydrolase, a glycosidase acting on glycosaminoglycans; and oxidoreductase/short chain dehydrogenase, potentially membrane bound and involved in d-gluconate metabolism [48]. Additionally, a putative uncharacterized protein (Accession: Q97NT9), present in the WT only is a protein encoded by a gene transcriptionally linked to *ply* and has been suggested to assist Ply transport from cytoplasm to cell wall [41]. As mentioned previously, FbpA is a SecA2-dependent protein in *L. monocytogenes* that acts as a chaperone for listeriolysin O [20]. It is possible one or more of the genes in the *ply* locus (e.g. SP_1924 which encodes Q97NT9) encodes proteins that act as a chaperone for Ply export.

Proteins that were present in both WT and Δ*secA2* mutant included typical cell surface proteins such as PspA (Q97T39) and PcpA (Q97NB5) (Tables S3 and S4). We were not able to detect tryptic peptides of PsrP in the CWP fraction of wild type strain TIGR4 (Table S3), suggesting that PsrP is tightly bound within the cell wall. However, we found 14 unique PsrP peptides in the CWP fraction unfiltered data, i.e. identified at >5% false discovery rate, that were present in WT but not in the Δ*sec*A2 mutant (Table 1). Recently it has been shown that pyruvate oxidase (and H_2_O_2_ production) contributes to Ply release in a lysis-independent manner [49]. However, pyruvate oxidase was present in similar amounts in WT and Δ*secA2* mutant cell walls or culture fluid (data not shown).

In summary, we present data suggesting that the accessory Sec system is involved in the activation of Ply in the cell wall compartment of *S. pneumoniae*. One possibility is that Ply becomes transiently modified in transition from cytoplasmic to cell wall compartments. Alternatively, Ply becomes tightly associated with a peptidoglycan precursor [18] that requires SecY2A2 function for activation of Ply. The mechanisms of transcriptional regulation of the *ply* locus and Ply transport across the cytoplasmic membrane are thus still not understood. However, evidence that the accessory Sec system is involved in export of several potential virulence factors, including Ply, provides further motivation for identifying molecules that might selectively block the accessory Sec translocase.

## Conflict of interest

The authors declare no conflicts of interest.
