# Supplementary material for: The accessory Sec system (SecY2A2) in Streptococcus pneumoniae is involved in export of pneumolysin toxin, adhesion and biofilm formation
Source: Microbes Infect. 2017 Jul-Aug;19(7-8):402–12. doi: 10.1016/j.micinf.2017.04.003 (PMC5526788; doi:10.1016/j.micinf.2017.04.003)
Supplement: Supplementary file 1 [file mmc1.pdf]

Supplementary data

Table S1. Bacteria and plasmids

**Strain or plasmid**	**Relevant characteristics**	**Source**
TIGR4	Wild type (serotype 4)	Laboratory stock
UB2312	Δ*psrP*::*ermAM* (Em^R)	T. J. Mitchell
UB2570	Δ*secA2*::*aad9* (Sp^R)	This study
UB2706	Δ*ply*::*cat* (Cm^R)	This study
UB2707	Δ*secA2*Δ*ply* (Sp^R,Cm^R)	This study
UB2717	Δ*ply*/pMSP*ply*$^+$ (Cm^R, Em^R)	This study
UB2719	Δ*secA2*Δ*ply*/pMSP*ply*$^+$ (Sp^R, Cm^R, Em^R)	This study
UB2768	Δ*ply*/pMSP*ply*$_{63}^+$ (Cm^R, Em^R)	This study
UB2769	Δ*secA2*Δ*ply*/pMSP*ply*$_{63}^+$ (Sp^R, Cm^R, Em^R)	This study
pGEM®-T	PCR product cloning vector (Ap^R)	Promega
pGEM®-Δ*secA2*::*aad9*	Contains *aad9* (with promoter and terminator) inserted between *asp3* up and *gtfA* down flanking regions of *secA2* (Sp^R)	This study
pGEM®-Δ*ply*::*cat*	Contains *cat* (with promoter) inserted between SP_1924 up and SP_1922 down flanking regions of *ply* (SP_1923) (Ap^R)	This study
pFW5	2.8 kb carrying *aad9* (Sp^R)	A. Podbielski
pR326	3.99 kb *bla*, *cat* (Ap^R Cm^R)	J-P. Claverys
pMSP7515	12.63 kb *prgB*, *ermAM*, *nisR*, *nisK* P*nisA* (Em^R) nisin-inducible *prgB*	G.M. Dunny
pMSP*ply* (denoted p*ply*)	9.8 kb *ermAM*, *nisR*, *nisK* P*nisA ply* (Em^R) nisin-inducible *ply*	This study
pMSP*ply63* (denoted p*ply63*)	9.8 kb *ermAM*, *nisR*, *nisK* P*nisA ply63* (Em^R) nisin-inducible *ply* (Ply:Thr_{63}Ala)	This study

Table S2. Primers used in this study

**Name**	**Primer sequence 5'---- >3'** [a,b]	**Target**
MBF7	CCTCAGCTTCCCTTATTAAA	*asp3-secA2*
MBR7	**CAATCACCTGCTTC**GGATCCCCTTCCCCGTATTCATCTC	*asp3-secA2*
MBF8	**AATACGGGGGAAGG**GGATCCGAAGCAGGTGATTGATAAAG	*secA2-gtfA*
MBR8	ATCCACTGTCACGCTAGTAG	*secA2-gtfA*
BamSpecF	CGCGGATCCGATCATATCATATATAATCTAGAAT	*aad9*
BamSpecR	GCGGGATCCGCCCATGGCGCGCTTACCAATTAGA	*aad9*
Up.PlyF	GAGAGGAGATGTTGTAGCTC	SP_1924-*ply*
Up.PlyR	**GTCCCAAGCC**GGATCCAGAGGCGACTGTCGTTGGTAGC	SP_1924-*ply*
Down.PlyF	**GACAGTCGCCTCT**GGATCCGGCTTGGGACAGAAATGGGC	*ply*-SP_1922
Down.PlyR	CAAACTAGACCATTATCTGC	*ply*-SP_1922
BamCatF2	CGCGGATCCGTTCAACAAACGAAAATTGGATAAAGT	*cat*
BamCatR	GCGGGATCCGGAGAAGTCGGTTCAGAAAAAG	*cat*
NcoIPlyF	CGCCCATGG**AGGAGGATGATAC**ATGGCAAATAAAGCAGTAAATGAC	*ply*
XhoIPlyR	GCGCTCGAGTTACTAGTCATTTTCTACCTTATCTTC	*ply*
PlyMutA.F[c]	**GCC**GCTACCAACGACAGTCGCCTCTATCCT	*ply*$_{63}$
PlyMutA.R[c]	TACAGAAATATCACTTGTATTTGTCGAC	*ply*$_{63}$

[a] Restriction sites are underlined.

[b] Sequence overhangs are in bold.

[c] 5' end phosphate modification.

Table S3. Top 100 hits of proteins identified in *S. pneumoniae* TIGR4 versus Δ*secA2* mutant (shaded grey) cell wall protein extracts. TIGR4 peptides are sorted highest to lowest number of unique peptide sequences.

Acc.[a]	pI[b]	Description	% Coverage[c]		PSM[d]		Peptides[e]	
			WT	Δ*secA2*	WT	Δ*secA2*	WT	Δ*secA2*
I6L8R4	6.13	β-galactosidase	62.52	44.33	321	128	111	71
Q97RY6	6.34	Serine protease	54.72	36.21	210	95	92	61
Q2MGH6	6.07	Endo-α-*N*-acetylgalactosaminidase	63.95	2.21	273	2	90	2
Q97NQ8	7.03	DNA-directed RNA polymerase subunit β	57.96	53.39	179	129	64	61
Q97RK7	4.93	Aminopeptidase N	77.48	84.55	291	246	59	65
Q97QP7	5.38	Immunoglobulin A1 protease	42.37	35.68	102	70	56	45
Q97NQ7	5.14	DNA-directed RNA polymerase subunit β	55.11	46.30	159	114	52	47
P64022	4.96	Elongation factor G	86.29	89.47	429	353	51	59
Q97NK0	6.61	Alcohol dehydrogenase	70.55	71.46	237	211	51	55
P95829	4.77	Chaperone protein DnaK	88.80	89.29	454	335	49	54
Q97SC6	5.25	Formate acetyltransferase	77.13	78.04	203	135	48	47
P29849	5.17	Maltodextrin phosphorylase	65.03	62.90	172	125	42	41
Q9L7Q2	5.25	Zinc metalloprotease zmpB	31.16	33.16	88	93	42	44
Q97SK0	5.08	Putative ATP-dependent Clp protease, ATP-binding subunit	69.90	74.18	155	125	41	48
P67580	4.98	Methionine-tRNA ligase	62.86	62.41	117	85	40	39
Q97QM4	5.48	Ribonucleoside-diphosphate reductase	63.42	55.35	101	58	40	31
Q54869	5.15	Arginine-tRNA ligase	74.42	74.07	116	84	39	36
Q54970	5.17	Pyruvate oxidase	71.40	73.43	361	293	39	46
Q97S90	5.31	Putative endo-β-*N*-acetylglucosaminidase	33.33	3.92	76	3	39	3
Q97Q48	5.15	Alanine-tRNA ligase	52.87	40.25	83	55	38	30
Q97S45	5.17	Valine-tRNA ligase	50.74	48.24	102	77	38	35
Q97RC5	5.15	Pyruvate kinase	77.64	72.65	279	207	37	37
Q97PH0	4.83	Endopeptidase O	68.10	67.46	154	133	37	42
Q97S57	8.84	Translation initiation factor IF-2	51.36	47.29	73	53	37	34
P64030	4.97	Elongation factor Tu	90.45	86.18	631	467	36	35
Q97SQ7	5.69	Putative alkaline amylopullulanase	40.47	22.50	64	22	36	18
Q97PD6	5.55	Protein translocase subunit SecA 1	52.81	46.12	88	46	35	29
P0CB61	5.00	Isoleucine-tRNA ligase	45.27	35.27	93	53	35	25
P0A335	4.84	60 kDa chaperonin	60.74	66.30	162	131	32	38
Q97PU5	4.82	Phosphoglucomutase	74.48	88.81	105	106	31	35
Q97SQ9	5.15	Glucosamine-fructose-6-phosphate aminotransferase	67.61	83.72	103	156	31	42
Q97SR1	5.07	Proline-tRNA ligase	57.37	63.53	67	67	31	31

Q97QS2	4.81	Enolase	85.71	91.94	481	466	30	37
P22976	5.22	Probable transketolase	75.99	76.44	123	117	30	33
Q97RF9	5.14	Ribosomal protein S1	68.75	69.25	155	121	30	31
P65887	5.69	Adenylosuccinate synthetase	66.36	66.82	89	84	30	30
Q97RC8	5.53	Xaa-Pro dipeptidyl-peptidase	45.57	44.65	57	52	30	29
Q97S89	4.98	Phosphoglycerate kinase	88.69	90.95	481	437	29	37
Q97QM7	4.72	Phosphoenolpyruvate-protein phosphotransferase	67.94	69.15	168	109	29	32
P64297	5.08	GMP synthase [glutamine-hydrolyzing]	65.58	69.23	93	85	29	28
Q97SJ2	5.50	Capsular polysaccharide biosynthesis protein Cps4K	66.99	65.04	71	63	29	28
Q97NA4	5.08	Arginine deiminase	60.39	46.45	125	32	29	16
Q97SS0	5.02	Leucine-tRNA ligase	42.74	51.86	70	50	29	26
Q97QE4	4.89	Carbamoyl-phosphate synthase large chain	39.22	30.15	58	42	29	25
Q97T93	5.36	Adenylosuccinate lyase	70.14	68.98	70	54	28	24
Q97PW6	5.00	Glycine-tRNA ligase β subunit	42.77	32.45	53	31	28	18
Q97SP5	5.21	Aminopeptidase C	61.04	67.12	112	81	27	29
Q97S34	4.73	Phenylalanine-tRNA ligase β subunit	44.44	37.45	60	36	27	22
Q97QX6	5.69	Phosphoenolpyruvate carboxylase	39.42	41.65	46	49	27	30
P65607	5.39	Ornithine carbamoyltransferase, catabolic	73.08	60.65	321	54	26	20
Q97T39	4.91	Pneumococcal surface protein A	47.45	43.01	117	56	26	22
P35594	5.69	ATP-dependent Clp protease ATP-binding subunit ClpE	38.96	33.38	44	30	26	20
Q97S28	5.10	Polyribonucleotide nucleotidyltransferase	38.53	41.52	87	62	26	26
P0A3B7	4.93	Elongation factor Ts	84.39	86.71	222	175	25	25
Q97N43	5.34	Inosine-5'-monophosphate dehydrogenase	65.24	68.09	80	44	25	25
Q97NL1	5.47	Glyceraldehyde 3-phosphate dehydrogenase	89.25	94.03	417	469	24	31
Q97NH4	5.45	Alcohol dehydrogenase, zinc-containing	86.93	89.20	92	78	24	25
I6L8U5	5.67	3-oxoacyl-[acyl-carrier-protein] synthase 2	81.02	73.48	82	50	24	20
Q97SI6	5.06	6-phosphogluconate dehydrogenase, decarboxylating	73.21	72.78	119	149	24	30
Q97N41	4.84	ABC transporter, ATP-binding protein	58.70	60.37	57	47	24	25
Q97PI4	5.40	Threonine-tRNA ligase	52.55	41.89	71	42	24	17
Q97RV5	4.91	Elongation factor Tu family protein	49.27	45.02	87	79	24	26
Q97SG9	4.50	Trigger factor	62.30	64.17	111	109	23	26
Q97SE7	5.29	Aspartyl/glutamyl-tRNA(Asn/Gln) amidotransferase subunit B	54.17	44.79	43	38	23	21

Q97PR0	5.06	Asparagine-tRNA ligase	46.98	45.41	66	48	23	22
Q97PK2	4.93	UDP-glucose 4-epimerase	85.55	86.73	87	68	22	23
Q97RS8	6.16	Lactate oxidase	83.33	84.92	167	101	22	25
Q97T85	5.02	Sugar isomerase domain protein AgaS	70.62	28.09	56	9	22	8
Q97QB4	5.47	Glutamate dehydrogenase	59.38	62.05	119	98	22	27
Q97RS9	5.49	Lysine-tRNA ligase	46.98	41.53	50	44	22	22
Q97P40	4.69	Putative general stress protein 24	92.57	92.57	484	119	21	20
P66565	10.2	30S ribosomal protein S4	84.73	88.18	90	77	21	24
Q97SP1	5.06	Alcohol dehydrogenase, zinc-containing	84.66	76.70	111	70	21	22
P0A3M9	5.22	L-lactate dehydrogenase	75.91	77.74	108	153	21	25
Q97QL3	4.92	Tagatose 1,6-diphosphate aldolase	69.94	60.12	93	38	21	18
Q97PL8	5.00	Oxidoreductase, pyridine nucleotide-disulfide, class I	64.84	50.46	69	31	21	16
P63413	5.24	Acetate kinase	63.64	63.64	55	46	21	21
I6L8V0	5.43	Capsular polysaccharide biosynthesis protein Cps4J	64.96	69.23	70	64	21	27
Q97PX1	5.12	NADH oxidase	54.03	60.78	146	108	21	23
Q97S86	5.05	Glutamine synthetase, type I	48.66	63.62	64	76	21	31
Q97R16	5.29	Serine hydroxymethyltransferase	54.31	61.24	43	47	21	23
Q97T35	5.90	Metallo-β-lactamase superfamily protein	49.19	39.53	41	30	21	18
Q97R51	5.12	Foldase protein PrsA	75.08	75.40	67	55	20	24
Q97NM1	5.66	Catabolite control protein A	79.76	72.62	57	35	20	17
Q97RI6	5.20	Phosphopentomutase	72.70	54.34	54	27	20	16
Q97SG0	5.08	Serine-tRNA ligase	67.22	53.30	51	38	20	17
Q97SE6	5.02	Glutamyl-tRNA(Gln) amidotransferase subunit A	64.96	62.09	59	58	20	21
Q97SF8	6.15	Aspartokinase	64.54	59.91	46	39	20	20
Q97PF9	4.81	Cell division protein FtsZ	56.56	57.28	63	52	20	22
O54537	5.66	Glucose-6-phosphate 1-dehydrogenase	57.78	50.30	34	31	20	19
Q97PW8	5.20	Putative oxidoreductase	62.56	45.65	61	28	20	14
Q97QD2	8.07	Signal recognition particle protein	54.30	52.39	45	34	20	19
Q97RB6	5.78	Putative transcriptional regulator	34.27	14.39	27	12	20	10

Q97QT1	5.35	Putative pullulanase	31.88	27.14	47	24	20	15
Q97N56	5.26	30S ribosomal protein S2	68.34	71.04	121	143	19	22
Q97S02	4.94	Dipeptidase	53.65	65.45	55	58	19	23
Q97NG0	5.14	Glucose-6-phosphate isomerase	56.35	52.34	108	83	19	17
Q97SP8	4.92	Aminopeptidase PepS	56.17	55.21	75	52	19	19
I6L8U0	6.18	UDP-*N*-acetylglucosamine 2-epimerase	51.02	42.64	65	42	19	16
Q97S60	4.97	N utilization substance protein A	48.68	42.06	43	41	19	17

[a] Protein accession number.
[b] Calculated isoelectric point.
[c] Percent of the protein sequence covered by identified peptides.
[d] Total number of identified peptide sequences (peptide spectrum matches) for the protein.
[e] Number of unique peptide sequences.

Table S4. Top 100 hits of proteins identified in *S. pneumoniae* TIGR4 versus Δ*secA2* mutant (shaded grey) culture supernatant extracts. TIGR4 peptides are sorted highest to lowest number of unique peptide sequences.

Acc.[a]	pI[b]	Description	% Coverage[c]		PSM[d]		Peptides[e]	
			WT	Δ*secA2*	WT	Δ*secA2*	WT	Δ*secA2*
Q9L7Q2	5.25	Zinc metalloprotease ZmpB	57.71	40.40	197	133	84	59
Q97RY6	6.34	Serine protease, subtilase family PrtA	38.32	19.07	146	61	71	35
Q97NK0	6.61	Alcohol dehydrogenase, iron-containing	54.47	58.55	130	125	44	49
Q97QS2	4.81	Enolase (Eno)	91.01	67.97	212	139	36	34
Q97T80	5.57	Zinc metalloprotease ZmpC	30.66	11.69	60	21	35	15
Q54970	5.17	Pyruvate oxidase	63.11	62.61	130	125	35	45
Q54869	5.15	Arginine-tRNA ligase	62.88	51.15	67	56	33	26
Q97SC6	5.25	Formate acetyltransferase	52.97	43.15	80	49	33	29
P64022	4.96	Elongation factor G	60.32	76.05	86	102	33	46
P95829	4.77	Chaperone protein DnaK	64.91	64.25	75	104	31	38
Q97RK7	4.93	Aminopeptidase N	44.22	35.38	57	35	30	21
Q97S89	4.98	Phosphoglycerate kinase	80.15	84.67	162	84	29	29
P64030	4.97	Elongation factor Tu (EF-Tu)	66.08	61.06	142	128	28	30
Q97QM4	5.48	Ribonucleoside-diphosphate reductase	46.31	25.31	40	24	26	14
Q97PH0	4.83	Endopeptidase O	55.40	41.11	50	46	26	22
P66565	10.15	30S ribosomal protein S4	90.64	93.60	100	73	26	30
Q97N55	6.07	Secreted 45 kd protein	84.69	81.63	73	88	26	32
P18791	5.06	Oligopeptide-binding protein AmiA	61.15	42.49	62	55	25	23
Q97NL1	5.47	Glyceraldehyde 3-phosphate dehydrogenase (GAPDH)	90.15	83.28	130	120	25	28
Q97RC5	5.15	Pyruvate kinase (PK)	54.29	55.29	61	59	25	28
Q97RF9	5.14	Ribosomal protein S1	61.50	68.00	67	75	25	33
Q97NB5	7.14	Choline binding protein PcpA	43.16	18.04	46	16	24	9
Q97T93	5.36	Adenylosuccinate lyase	59.03	44.21	36	28	24	15
P65887	5.69	Adenylosuccinate synthetase	57.01	50.23	55	37	24	21
Q97NQ8	7.03	DNA-directed RNA polymerase subunit β	23.84	20.73	34	30	24	22

Q97NQ7	5.14	DNA-directed RNA polymerase subunit β	26.52	23.28	44	35	24	24
Q97NA4	5.08	Arginine deiminase	49.88	30.32	60	16	23	11
Q97P40	4.69	Putative general stress protein 24	93.56	64.85	106	37	23	17
Q97SI6	5.06	6-phosphogluconate dehydrogenase	59.49	53.38	59	57	23	24
Q97SQ9	5.15	Glucosamine-fructose-6-phosphate aminotransferase	53.99	51.50	49	43	23	25
Q97N56	5.26	30S ribosomal protein S2	71.04	76.45	98	76	23	28
P0A335	4.84	60 kDa chaperonin	42.41	56.30	45	56	23	30
Q2MGH6	6.07	Endo-α-*N*-acetylgalactosaminidase	19.07	7.36	34	13	22	8
Q97N37	6.96	Serine protease	71.76	53.44	48	23	22	14
I6L8V0	5.43	Capsular polysaccharide biosynthesis protein Cps4J	63.25	57.26	42	28	22	21
Q97Q36	4.81	Putative phosphate transport system regulatory protein PhoU	87.10	54.84	75	19	21	10
Q97RH0	5.52	Lipoprotein	65.43	44.29	51	21	21	11
P0A4G2	5.43	Manganese ABC transporter substrate-binding lipoprotein	68.93	69.90	63	51	21	17
Q97NE3	5.67	Tyrosine-tRNA ligase	59.09	54.31	48	39	21	17
P65607	5.39	Ornithine carbamoyltransferase, catabolic	65.68	75.44	74	45	21	23
Q97R51	5.12	Foldase protein PrsA	74.12	62.62	49	34	20	16
P0A3Y3	5.31	2,3-bisphosphoglycerate-dependent phosphoglycerate mutase	75.22	68.26	73	49	20	18
Q97SV1	9.06	50S ribosomal protein L5	88.89	88.89	112	68	20	18
I6L8U5	5.67	3-oxoacyl-[acyl-carrier-protein] synthase 2	67.88	65.45	46	34	20	19
P0A3B7	4.93	Elongation factor Ts	73.41	50.58	120	63	20	21
P59213	5.22	Maltose/maltodextrin-binding protein	58.39	53.90	48	44	20	24
Q97SG9	4.50	Trigger factor	46.60	69.79	51	77	20	29
Q2MGF6	6.93	Lysozyme	38.16	19.59	31	12	19	9
Q97SJ2	5.50	Capsular polysaccharide biosynthesis protein Cps4K	47.43	38.88	43	28	19	16
P66050	5.12	50S ribosomal protein L10	87.35	77.71	149	52	19	18
Q97PU5	4.82	Phosphoglucomutase	47.55	47.03	43	41	19	21

P0A4S1	5.10	Fructose-bisphosphate aldolase	65.19	75.77	150	88	19	23
Q97PF9	4.81	Cell division protein FtsZ	45.58	47.49	35	55	19	23
Q97S45	5.17	Valine-tRNA ligase	26.50	13.93	30	15	18	8
Q97NG0	5.14	Glucose-6-phosphate isomerase	52.12	39.42	45	23	18	12
Q97SP1	5.06	Alcohol dehydrogenase	63.72	57.23	48	39	18	15
Q97T12	5.26	ABC transporter	72.10	68.48	46	36	18	16
P66581	9.52	30S ribosomal protein S5	84.15	84.15	90	67	18	18
Q97RS8	6.16	Lactate oxidase	68.78	68.78	55	44	18	20
Q97SP2	5.29	PTS system, mannose-specific IIAB components	61.45	58.43	50	49	18	23
Q97QB4	5.47	Glutamate dehydrogenase	49.33	45.54	40	49	18	26
P29849	5.17	Maltodextrin phosphorylase	31.25	25.00	29	18	17	11
Q97PX1	5.12	NADH oxidase	48.80	19.17	39	18	17	11
Q97QE7	4.86	Choline kinase	60.31	40.46	32	22	17	13
Q97SU7	9.52	50S ribosomal protein L6	80.34	83.15	50	31	17	15
Q97NH4	5.45	Alcohol dehydrogenase, zinc-containing	61.08	50.00	40	32	17	15
P35592	5.16	Oligopeptide-binding protein AliA	36.36	32.73	28	28	17	17
Q97SN5	9.95	50S ribosomal protein L13	89.86	91.89	62	75	17	19
P67580	4.98	Methionine-tRNA ligase	32.93	35.04	30	43	17	21
Q97QL3	4.92	Tagatose 1,6-diphosphate aldolase	58.59	29.45	27	12	16	8
Q97RS9	5.49	Lysine-tRNA ligase	29.64	21.77	31	21	16	9
Q97R16	5.29	Serine hydroxymethyltransferase	43.78	33.01	26	15	16	11
Q97Q48	5.15	Alanine-tRNA ligase	23.17	16.40	25	16	16	11
P64297	5.08	GMP synthase	36.92	32.31	26	24	16	12
Q97S02	4.94	Dipeptidase	35.41	30.04	25	22	16	12
P22976	5.22	Probable transketolase	40.58	39.97	38	31	16	17
P0A4C3	9.70	30S ribosomal protein S3	68.20	71.89	59	46	16	19
Q97SV2	10.68	50S ribosomal protein L2	62.09	72.20	51	74	16	24
Q97T85	5.02	Sugar isomerase domain protein AgaS	44.85	19.07	20	5	15	5
Q97NQ0	5.16	Aspartate-ammonia ligase	65.15	40.91	28	12	15	8
Q97PM1	5.99	GTP-sensing transcriptional pleiotropic repressor CodY	56.49	35.88	36	16	15	9

Q97PK2	4.93	UDP-glucose 4-epimerase	67.26	34.22	34	16	15	9
Q97PN2	4.74	Non-heme iron-containing ferritin	87.64	51.69	62	22	15	9
Q97R14	5.64	Putative uncharacterized protein	50.00	38.89	31	18	15	10
Q97R09	5.54	Iron-compound ABC transporter, iron compound-binding protein	62.17	44.87	33	20	15	10
Q97T11	5.63	Lipoprotein	57.75	50.35	27	16	15	11
Q97SP5	5.21	Aminopeptidase C	41.67	29.28	31	18	15	11
Q97T39	4.91	Pneumococcal surface protein A	28.49	15.59	28	19	15	11
Q97RI6	5.20	Phosphopentomutase	51.61	50.62	32	27	15	13
Q97NG1	5.00	Glutamate-tRNA ligase	32.30	35.19	22	23	15	14
P66708	4.75	DNA-directed RNA polymerase subunit α	48.55	43.41	30	23	15	15
Q97N43	5.34	Inosine-5'-monophosphate dehydrogenase	46.54	38.41	24	24	15	15
Q97QM7	4.72	Phosphoenolpyruvate-protein phosphotransferase	35.70	31.89	42	46	15	20
Q97QP6	8.81	Ribosome biogenesis GTPase A	47.35	12.72	22	7	14	5
Q97PW6	5.00	Glycine-tRNA ligase βsubunit	21.68	12.83	24	11	14	7
Q97S60	4.97	N utilization substance protein A	39.42	24.07	19	11	14	7
Q97PQ7	6.20	Conserved domain protein	70.33	63.16	34	18	14	10
Q97SU1	5.10	Adenylate kinase	75.00	68.87	34	29	14	11
Q97PD6	5.55	Protein translocase subunit SecA 1	21.15	15.89	17	14	14	11
Q97T63	5.66	ABC transporter, substrate-binding protein	33.81	28.31	23	20	14	11

[a] Protein accession number.
[b] Calculated isoelectric point.
[c] Percent of the protein sequence covered by identified peptides.
[d] Total number of identified peptide sequences (peptide spectrum matches) for the protein.
[e] Number of unique peptide sequences.

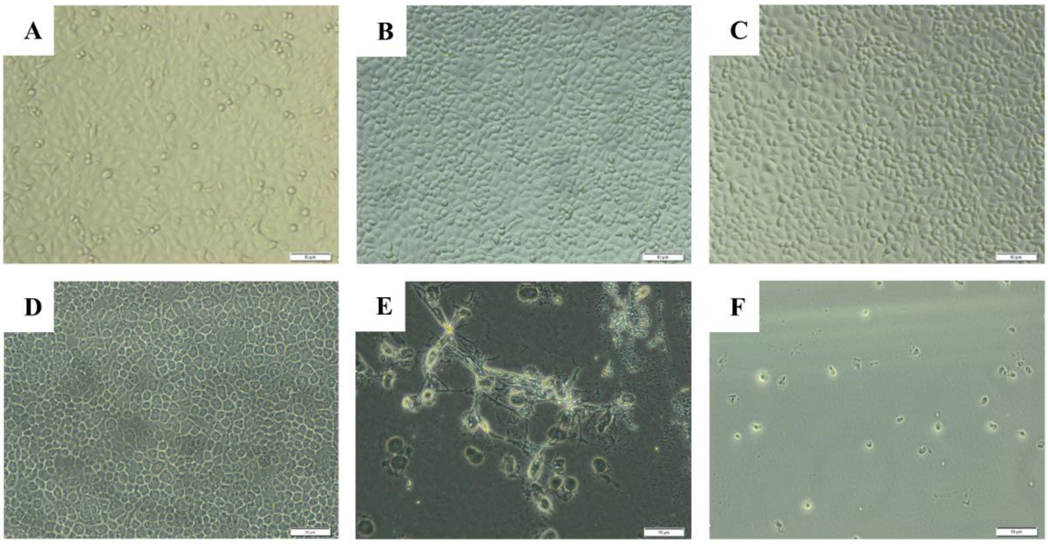

Fig. S1. A549 epithelial cells during the A549 association assay. Images of A549 cells during the association assay were taken on an inverted microscope to monitor the state of the epithelial layer at each stage of the assay. (A) Confluent layer of A549 cells with a density of 2 x 10^5 cells. A549 cells were infected with (B) and without bacteria (C) with an incubation period of 2 h. (D) A549 cells and bacteria were treated with cold dH_2O for 20 min to detach A549 cells. This step was repeated for a further 20 min (E + F) to ensure majority of A549 cells were detached. Scale bar = 10 µm.

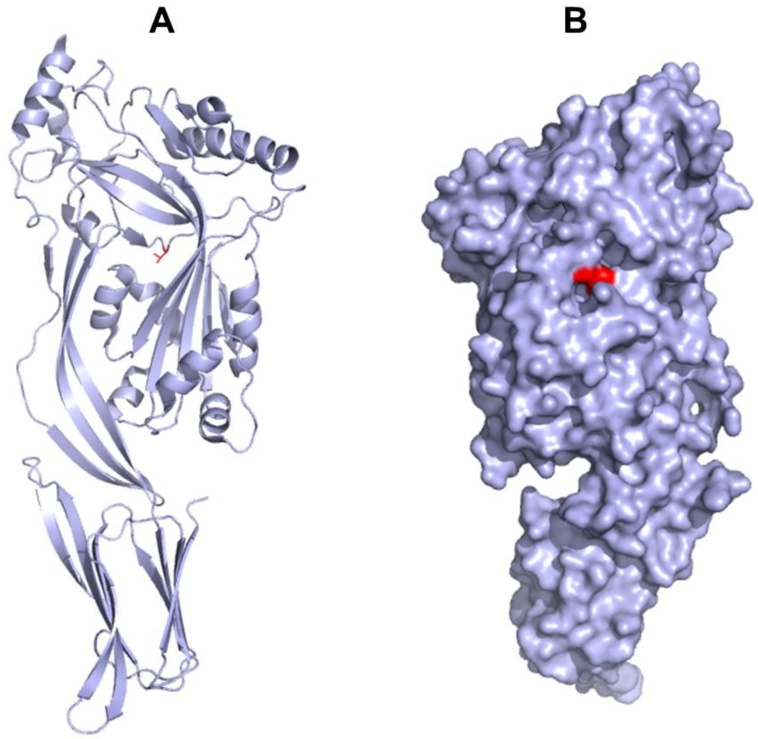

Fig. S2. Three dimensional models of pneumolysin protein structure. Ribbon (A) and surface (B) representations of pneumolysin were manipulated on PyMOL. The crystal structure of pneumolysin from *Streptococcus pneumoniae* D39 (PDB code: 5CR6) was used as a template. The position of residue 63, a threonine residue, is highlighted in red. This residue was replaced by alanine residue in Ply63.

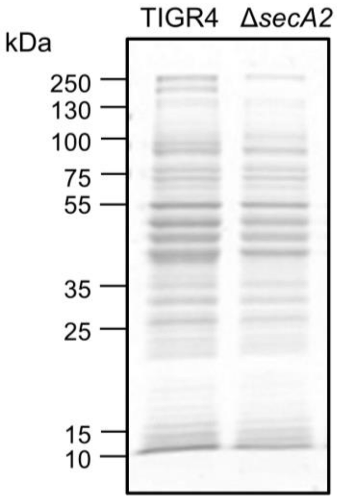

Fig. S3. SDS-PAGE profiles of cell wall protein (CWP) fractions. *S. pneumoniae* TIGR4 wild type and Δ*secA2* mutant cells were incubated with mutanolysin in osmotically-supported medium to release cell wall-associated proteins. These were separated by electrophoresis, and stained with Coomassie (as shown). Samples were also subjected to brief SDS-PAGE, in-gel tryptic digestion and peptides analyzed by LC-MS/MS.
